# Association of GrimAge DNA methylation components and 2-year mortality in the Health and Retirement Study

**DOI:** 10.1093/geroni/igab046.2531

**Published:** 2021-12-17

**Authors:** Helen Meier, Colter Mitchell, Eileen Crimmins, Bharat Thyagarajan, Jessica Faul

**Affiliations:** 1 University of Michigan, Ann Arbor, Michigan, United States; 2 University of Michigan, University of Michigan, Michigan, United States; 3 University of Southern California, Los Angeles, California, United States; 4 University of Minnesota, Minneapolis, Minnesota, United States

## Abstract

DNA methylation (DNAm) patterns related to age and aging phenotypes (i.e., epigenetic clocks) are of growing interest as indicators of biological age and risk of negative health outcomes. We investigated associations between the components of GrimAge, an epigenetic clock estimated from DNAm patterns for seven blood protein levels and smoking pack years, and 2-year mortality in the Health and Retirement Study (HRS) to determine if any of the DNAm subcomponents were driving observed associations. A representative subsample of individuals who participated in the HRS 2016 Venus Blood Study were included in this analysis (N=3430). DNAm was measured with the Infinium Methylation EPIC BeadChip. Deaths that occurred between 2016 and 2018 contributed to 2-year mortality estimates (N=159, 4.5% of the sample). Weighted logistic regression estimated the association first between GrimAge and 2-year mortality and second between the DNAm subcomponents and 2-year mortality. All models were adjusted for age, sex, race/ethnicity, education, current smoking status, smoking pack years and cell composition of the biological sample. The average GrimAge for participants with and without 2-year mortality was 77 years 68 years respectively. A one-year increase in GrimAge was associated with 17% higher odds of 2-year mortality (95% CI: 1.16, 1.17). Two of the seven DNAm blood protein subcomponents of GrimAge (TIMP metallopeptidase inhibitor 1, adrenomedullin) and DNAm smoking pack years were associated with 2-year mortality and DNAm smoking pack years appeared to drive the overall GrimAge association with 2-year mortality. GrimAge was a better predictor of 2-year mortality than the DNAm subcomponents individually.

